# Utilization of Sexual Reproductive Health Services Among Youths in Malaysia: A Cross-Sectional Study Applying Andersen’s Behavioral Model of Healthcare Utilization

**DOI:** 10.7759/cureus.60230

**Published:** 2024-05-13

**Authors:** Rowena John, Nur Afiqah Mohd Salleh, Nik Daliana Nik Farid

**Affiliations:** 1 Department of Social and Preventive Medicine, Faculty of Medicine, Universiti Malaya, Kuala Lumpur, MYS; 2 Centre for Population Health, Department of Social and Preventive Medicine, Faculty of Medicine, Universiti Malaya, Kuala Lumpur, MYS

**Keywords:** malaysia, reproductive health services, youth health, sexual and reproductive, service utilization

## Abstract

Background

The sexual well-being of youths is crucial as it establishes the foundation for their sexual health throughout their lives. Malaysia’s Ministry of Health (MOH) mainly delivers sexual reproductive health (SRH) services. Besides MOH, the National Population Family Development Board (NPFDB), under the purview of the Ministry of Women, Family and Community Development and Federation of Reproductive Health Association Malaysia, works closely with MOH to ensure the delivery of SRH information and services. Despite the availability of SRH services in Malaysia, it is uncertain whether youths are aware of and utilize these services. This study aims to identify factors that affect the utilization of SRH services among youths aged 18-24 years in Malaysia.

Methodology

This web-based, cross-sectional study was conducted from March 2022 to June 2022 using a self-administered pre-tested questionnaire. Andersen’s Behavioral Model of Health Service Utilization was used to identify the variables included in the survey. Bivariate and multivariate logistic regression models were used to determine factors significantly associated with the utilization of SRH services. Adjusted odds ratio (AOR) and 95% confidence interval (CI) with a p-value <0.05 were considered to denote statistical significance.

Results

A total of 617 youths aged 18-24 years participated in the survey. Only 20.4% (n = 126) of youths had visited SRH services in their entire life, and only 8.4% (n = 52) of youths had visited SRH services in the past year. Predisposing factors such as age, marital status, exposure to SRH information from family and governmental agencies such as the NPFDB, enabling factors such as availability and comfort of SRH services, and need factors such as youths diagnosed with SRH-related diseases were significantly associated with SRH utilization. The older age group (20-24 years old) was more likely to utilize SRH services compared to the 18-19-year age group (AOR = 1.634, 95% CI = 1.041, 2.564, p = 0.033). Married participants were three times more likely to utilize SRH services than single participants (AOR = 2.910, 95% CI = 1.356, 6.249, p = 0.006). Participants who vaped had more odds of utilizing SRH services (AOR = 1.793, 95% CI = 1.014, 3.174, p = 0.045) The group of participants exposed to information on SRH from family had more odds of utilizing SRH service than those who did not receive information on SRH from the family (AOR = 1.964, 95% CI = 1.229, 3.138, p = 0.005). Likewise, participants who received SRH information from governmental agencies were more likely to utilize SRH services (AOR = 1.929, 95% CI = 1.202, 3.095, p = 0.006). Enabling factors that were associated with SRH utilization were the availability of services, described as self-buying medicine in pharmacies (AOR = 1.830, 95% CI = 1.184, 2.855, p = 0.007), and the comfortability of services (AOR = 1.928, 95% CI = 1.250, 2.974, p = 0.003). Youths who were diagnosed with SRH diseases (need factor) were four times more likely to utilize SRH services (AOR = 4.490, 95% CI = 1.935, 10.410, p < 0.001).

Conclusions

There is generally poor SRH service utilization and awareness among youths in Malaysia, which could be improved. The findings of this study can be used to influence SRH providers to offer a more age-targeted awareness program to meet the various SRH needs of youths.

## Introduction

Sexual reproductive health (SRH) services include antenatal, delivery, and postpartum care services, as well as information and access to counseling, health education, family planning services, human immunodeficiency virus (HIV) screening, and diagnosis and treatment of sexually transmitted diseases [1]. Globally, around 80% of the estimated 1.21 billion youths between the ages of 15 and 24 lack access to necessary reproductive health care services, making SRH a significant public health concern [2].

One of society’s most important resources is its youth. The World Health Organization and United Nations Educational, Scientific and Cultural Organization define those between the ages of 15 to 24 years as youths [3]. The Society of Adolescent Health and Medicine has found, through an analysis of more than 40 publications in the United States, that individuals between the ages of 18 and 25 years have higher rates of mortality and unintended pregnancies. Additionally, they have lower access to healthcare services than younger individuals aged 10-17 years and older individuals aged 26-30 years [4].

Youth-centric SRH services are reported to be underutilized and continue to experience many challenges. At an individual level, youths frequently hide their SRH issues and do not seek the necessary medical attention. Additionally, inadequate knowledge about SRH, limited financial resources, or unfavorable attitudes among healthcare professionals further restrict youth’s access to SRH services [5]. Collectively, these have led to vulnerabilities towards sexually transmitted infections (STIs), HIV/acquired immunodeficiency syndrome (AIDS), maternal and child mortality, unplanned pregnancies, unsafe abortions, reduced use of contraception, sexual abuse (including rape), and female genital mutilation [6]. In Malaysia, sex before marriage, unplanned pregnancies, STIs, and unsafe abortions are significant issues affecting youths [7]. Between 2005 and 2015, the reported rates of premarital sex among those aged 12 to 24 years increased, ranging from 1.3% to 12.6% [8].

The delivery of SRH services in Malaysia is covered by not only the Ministry of Health (MOH) but also the National Population Family Development Board (NPFDB), under the purview of the Ministry of Women, Family and Community Development, and the Federation of Reproductive Health Association, Malaysia [8]. Most government primary care clinics have dedicated adolescent health clinics. Other programs include school-based health services by the MOH and the Teen Adolescent Centre, known as “KafeTeen,” managed by NPFDB. NPFDB 2021 annual report showed that 52,923 young people aged 13-24 years had been involved in their Teen Adolescent Centres, known as “KafeTeen” programs, in various states in Malaysia. Currently, there are 18 “KafeTeen” centers in Malaysia, which provide support services to young people in multiple aspects. A total of 2,868 young people aged 13-24 years have been referred for SRH-related issues [9].

Although there is availability of various SRH providers in Malaysia, less is known about SRH service awareness and factors affecting the utilization of SRH services among youths in Malaysia from the perspective of youths. This study aimed to determine the proportion of Malaysian youths aware of the availability of SRH services and who utilized these services and identify factors that affect service utilization. This study focuses on youths aged 18-24 years who are likely to be of the ages that require and seek SRH information and services and can decide on their health status without parental consent. This particular age group (ages 18 to 24) was chosen as the study population as it is a phase of life where individuals are expected to achieve financial independence, form romantic partnerships, become parents, and take on responsible roles as active and contributing members of society. Generally, this age group tends to seek their sexual partner as their life partner for marriage [10]. This age group primarily attends school, college, foundation courses, matriculation, and pre-university and may be exposed to early working life. Young people have poor levels of sexual health education and restricted access to SRH services, which has been associated with a higher incidence of sexual risk behaviors, unplanned pregnancies, and STIs [6].

Andersen’s Healthcare Utilization Model was used to conceptualize the potential factors influencing the utilization of SRH services through the following three key domains: predisposing factors, enabling factors, and need factors. This widely used model identifies factors affecting healthcare utilization. Predisposing factors include demographic characteristics of age, sex, and social factors (e.g., ethnicity and social relation). Predisposing factors exist before the onset of illness and relate to an individual’s propensity to seek help. Meanwhile, enabling factors are known as inhibiting factors and typically focus on the factors associated with accessibility and affordability. Lastly, need factors refer to a person’s perceived need for health services [11]. The model was applied to explore why youths use SRH healthcare services or otherwise assess inequality in youth’s access to health services, as well as aid in the creation of policies that will allow for equitable access to care for youths.

## Materials and methods

Study design and duration

This was a web-based, cross-sectional study performed among the general population of Malaysian youths aged 18-24 years who lived in Malaysia. Malaysia is an Asian country situated in Southeast Asia. Malaysia is partitioned by the South China Sea into two main regions, namely, Peninsular Malaysia, encompassing 11 states and two federal territories, and East Malaysia, consisting of two states and one federal territory. The study was conducted from March 2022 to June 2022.

Study population

Inclusion criteria

Malaysian youths aged 18-24 years who could read and write in English and Malay, who were residing in Malaysia, and who had access to the Internet were included in this study.

Exclusion Criteria

Non-Malaysians/foreigners and those who refused to participate in the study were excluded from the study.

Sampling technique and data collection method

An online survey was conducted using convenience sampling. Considering the data collection during the COVID-19 pandemic, this sampling was used as it is cost-effective, less time-consuming, and can easily reach the target population while adhering to the movement control order protocols. This study used a cross-sectional, web-based survey in which self-administered online questionnaires were published on social media platforms, including Facebook, Instagram, WhatsApp, and Twitter. Malaysian youths aged 18-24 years living in Malaysia were invited to participate in the survey. The questionnaire was uploaded and available online on all the above-mentioned social media platforms from March 2022 to June 2022. Respondent’s eligibility criteria were set to those aged 18-24 years living in Malaysia. Online consent was obtained before the commencement of the online questionnaire. To limit respondent bias, the first item was screening and necessary questions requesting if the respondent fulfilled the age, citizenship, and current country of residence and consented to participate in the survey. If the participants did not fulfill the inclusion criteria, they were immediately directed to the final part of the survey, saying they were not eligible to participate.

Sample size calculation

The sample size was calculated using EpiInfo version 7, considering the proportion (p) of the prevalence of youths who sought treatment or advice from healthcare providers to be 24.5% [12]. The assumptions of a 95% confidence level (level of significance), 5% margin of error, and 20% non-response were used to determine the sample size. Accordingly, the total sample size was calculated to be 617.

Data collection tool

The questionnaires were close-ended, adapted by reviewing the literature, and suited to the local situation. The questionnaire was adapted from an instrument developed by the United Nations Population Fund (UNFPA) to assess the knowledge, attitude, and utilization of health services and sexual behaviors among youths [13]. In addition, a local study had previously translated and pre-tested the questionnaire using the UNFPA instrument [5].

The questionnaire was first prepared in English and then translated to Bahasa Malaysia and back into English to ensure consistency between the two versions. The questionnaire underwent face validation with seven panel experts from various SRH providers in Malaysia who had more than 15 years of experience in their field. The questions underwent face validation and content validation. All questions were combined and arranged according to the predisposing, enabling, and need factors based on Andersen’s behavioral Model of Healthcare Utilization, with a few adjustments for practicality. A pilot study was also conducted among 40 participants to test the reliability of the questions.

Study variables

Variables were chosen based on a literature review of studies on SRH utilization [14-16]. The dependent variable was the utilization of SRH services (Yes/No response). Services related to SRH utilization were defined by considering the usage of at least one SRH service by youths. These services included counseling, provision of information and health education, family planning services, HIV counseling and testing, pregnancy testing, diagnosis and treatment of STIs, abortion, and antenatal and delivery care [14].

The independent variables were predisposing factors (age, gender, marital status, education, religion, ethnicity, parental education, marital status of parents, occupation, high-risk behaviors, sexual activity trends, and knowledge of SRH diseases); enabling factors (awareness of SRH services, availability of SRH services, source of income, costing, source of information, comfort of SRH services); and need factors (diagnosed with SRH-related diseases).

Data analysis

The data were analyzed using Jamovi version 2.3.15. Descriptive statistics (frequency, mean, and standard deviation (SD)) were used to describe the characteristics of the study population. The categorical variables were summarized using frequencies and percentages (%), whereas continuous variables were presented using mean and SD. Comparison between the two groups was analyzed using the chi-square test for categorical variables depending on the expected values in the cells and the independent t-test for continuous variables. The variables that were significant at the <0.25 level in the bivariate analysis were included in the multiple logistic regression to assess the relationship between SRH utilization and its associated factors, as these factors could be potential confounders. Therefore, including the confounders could provide better estimation in a regression analysis. In the final model, only significant variables (p < 0.05) were included. Results were presented as an adjusted odds ratio (AOR) with a 95% confidence interval (CI).

Ethical considerations

The study was approved by the Universiti Malaya Medical Ethics Committee (UMREC) with the reference number UM. TNC2/UMREC_1457.

## Results

Characteristics of respondents

In total, 617 eligible participants aged 18-24 years from 13 states and three federal territories in Malaysia participated in the survey. Participants were from the northern, central, southern, and east coasts, as well as Sabah and Sarawak. The overall mean age of the participants was 20 years old (SD = ±1.88), of whom 365 (58.2%) were females, and 252 (41.8%) were males. Table 1 presents the characteristics of participants in this study. Most participants were Malays (73.1%) and were studying (82.7%). Most respondents were from Malaysia’s central region (61.3%). More than 50% of participants were unaware of SRH services, only 20.4% utilized SRH services throughout their lives, and 8.4% utilized SRH services in the past year.

**Table 1:** Characteristics of respondents in the web-based survey on SRH service utilization among youths aged 18–24 years, Malaysia, 2022 (n = 617). SRH = sexual reproductive health

Parameters	Category	Number (n)	Percentage (%)
Age (years)	18–19	252	41.8
	20–24	365	58.2
Gender	Male	252	41.8
	Female	365	58.2
Marital status	Single	581	94.0
	Married	36	6.0
Religion	Islam	469	76.0
	Buddhism	49	7.6
	Hinduism	36	5.8
	Christianity	57	9.2
	Others	6	1.01
Ethnicity	Malay	451	73.1
	Chinese	72	11.7
	Indian	53	8.6
	Others	41	6.6
Currently studying	Yes	510	82.7
	No	107	17.3
Residence	Northern Region	73	11.8
	Central Region	378	61.3
	Southern Region	95	15.4
	East Coast	25	4.1
	Sabah/Sarawak	46	7.5
Awareness of SRH services	Yes	214	34.7
	No	403	65.3
Utilized SRH services in the entire life	Yes	126	20.4
	No	491	79.6
Utilized SRH services in the past one year	Yes	52	8.4
	No	565	91.6

Table 2 demonstrates the associations between several individual components of predisposing factors, enabling factors, and need factors with SRH utilization. Age, gender, marital status, participants with a history of vaping and alcohol, and receiving information on SRH from friends, family, and NPFDB were associated with SRH utilization (p < 0.25).

**Table 2:** Bivariate analyses of factors associated with SRH service utilization among youths aged 18-24 years, Malaysia, 2022 (n = 617). P-value < 0.25. SRH = sexual reproductive health; NPFDB = National Population Family Development Board

Variable		Utilization of SRH services, n (%)		Chi-square value	P-value
		Yes	No		
Predisposing factors					
Age (years)	18–19	37 (5.9)	215 (34.8)	8.63	0.030
	20–24	89 (14.4)	276 (44.7)		
Gender	Male	45 (7.2)	134 (21.7)	3.45	0.060
	Female	81 (13.1)	357 (57.9)		
Marital status	Single	15 (2.4)	21 (3.4)	10.6	0.001
	Married	111 (18.0)	470 (76.2)		
Vaping	Yes	25 (4.1)	65 (10.5)	3.51	0.061
	No	101 (16.3)	426 (69.0)		
Alcohol	Yes	23 (3.7)	56 (9.1)	4.21	0.040
	No	103 (16.7)	435 (70.5)		
Source of information					
Friends	Yes	90 (14.5)	279 (45.2)	8.90	0.003
	No	36 (5.8)	212 (34.4)		
Healthcare workers	Yes	97 (15.7)	294 (47.6)	12.6	<0.001
	No	29 (4.7)	197 (31.9)		
NPFDB	Yes	43 (6.9)	85 (13.8)	17.2	<0.001
	No	83 (13.4)	406 (65.8)		
Family	Yes	90 (14.6)	292 (47.3)	6.08	0.014
	No	36 (5.8)	199 (32.3)		
Enabling factors					
Awareness of SRH services	Yes	55 (8.9)	159 (25.7)	5.62	0.018
	No	71 (11.5)	332 (53.8)		
Availability of SRH services					
Self-buy at pharmacies	Yes	53 (8.6)	138 (22.4)	9.14	0.003
	No	73 (11.8)	353 (57.2)		
Comfortable in utilizing SRH services	Yes	82 (13.3)	236 (38.2)	11.6	<0.001
	No	44 (7.1)	255 (41.3)		
Need factor					
SRH-related disease diagnosis	Yes	15 (2.4)	12 (1.9)	21.4	<0.001
	No	111 (18.0)	479 (77.6)		

**Figure 1:**
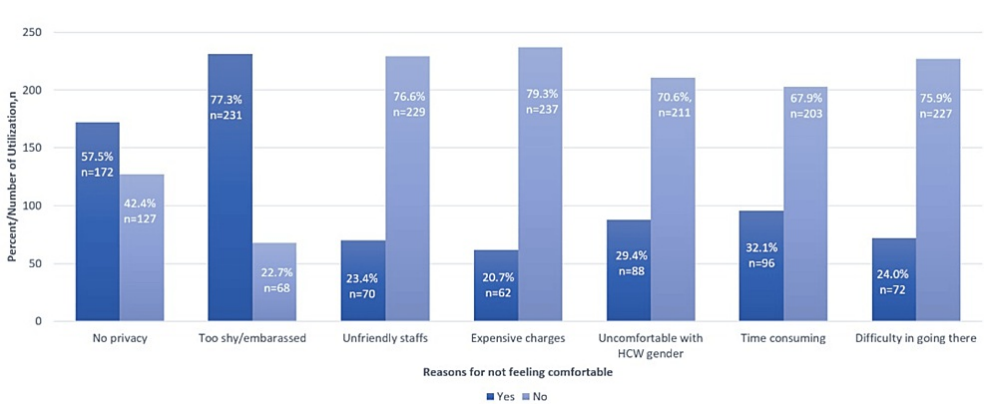
Reasons for not feeling comfortable in utilizing SRH services (n = 299). HCW = Healthcare worker; SRH = sexual reproductive health

Figure 1 illustrates various reasons why participants did not feel comfortable utilizing SRH services. Nearly half of them, around 299 (48.5%) participants, expressed discomfort using these services. A total of 231 (77.3%) participants reported being shy, and 172 (57.5%) participants reported no privacy.

Factors affecting SRH utilization

Findings from the logistic regression analyses (Table 3) indicate that predisposing factors such as age, marital status, and exposure to information from family and government agencies such as NPFDB were significantly associated with SRH utilization. The older age group (20-24 years old) was more likely to utilize SRH services than the 18-19-year age group (AOR = 1.641, 95% CI = 1.041, 2.579, p = 0.033). Married participants were three times more likely to utilize SRH services than single participants (AOR = 2.910, 95% CI = 1.356, 6.249, p = 0.006). Participants who vaped were more likely to utilize SRH services (AOR = 1.793, 95% CI = 1.014, 3.174, p = 0.045) than those who did not vape. The group of participants who had obtained information on SRH from family members were more likely to utilize SRH services than those who did not (AOR = 1.964, 95% CI = 1.229, 3.138, p = 0.005). Likewise, participants who received SRH information from governmental agencies such as NPFBD were more likely to utilize SRH services (AOR = 2.027, 95% CI = 1.257, 3.269, p = 0.004) than those who did not.

**Table 3:** Multiple logistic regression of factors of Andersen’s Healthcare Utilization Model associated with SRH services utilization among youths aged 18-24 years, Malaysia, 2022 (n = 617). Final model: Nagelkerke R^2^ = 0.169, Cox and Snell = 0.108, McFadden = 0.133, AUC predictive measures = 0.722. Multicollinearity was checked but not found. As all VIF values for the beta-coefficient were less than 5, there is evidence of no multicollinearity among independent variables. P-values <0.05 are considered statistically significant. OR = odds ratio; CI = confidence interval; R = reference group; SRH = sexual reproductive health; AUC = area under the curve; VIF = variance inflation factor

Factors		β	Crude OR (95% CI)	β	Adjusted OR (95% CI)	P-value
Age (years)	18–19		R			
	20–24	0.628	1.87 (1.23, 2.86)	0.51	1.64 (1.04, 2.58)	0.033
Marital status	Single		R			
	Married	1.11	3.02 (1.511, 6.06)	1.07	2.91 (1.36, 6.25)	0.006
Vaping	No		R			
	Yes	0.48	1.62 (0.97, 2.70)	0.58	1.79 (1.01, 3.17)	0.045
Received information from NPFDB	No		R			
	Yes	0.91	2.47 (1.60, 3.83)	0.70	2.03 (1.26, 3.27)	0.004
Received information from family	No		R			
	Yes	0.53	1.70 (1.11, 2.61)	0.69	1.96 (1.23, 3.14)	0.005
Availability of services: self-buy at pharmacies	No		R			
	Yes	0.62	5.39 (0.90, 2.47)	0.61	1.83 (1.18, 2.86)	0.007
Diagnosed with SRH-related diseases	No		R			
	Yes	1.69	5.39 (0.89, 2.47)	1.50	4.49 (1.94, 10.41)	<0.001
Comfortable in utilizing SRH services	No		R			
	Yes	0.70	2.01 (2.93, 1.11)	0.66	1.93 (1.25, 2.97)	0.003

Enabling factors associated with SRH utilization included the availability of services, described as self-buying medicine in pharmacies, and the participants’ comfort in utilizing the services. Participants who self-bought medicines in pharmacies were more likely to utilize SRH services (AOR = 1.830, 95% CI = 1.184, 2.855, p = 0.007) compared to those who did not. Participants who were comfortable with SRH services were two times more likely to utilize SRH services (AOR = 1.928, 95% CI = 1.250, 2.974, p = 0.003) than those who did not.

The needs factor that was associated with SRH utilization was having a diagnosis of SRH-related diseases. Youths who were diagnosed with SRH-related diseases were four times more likely to utilize SRH services (AOR = 4.490, 95% CI = 1.935, 10.410, p < 0.001).

The goodness of fit was assessed using receiver operating characteristics curves and the area under the curve (AUC). The AUC values were between 0 and 1; an AUC of 0.9-1.0 is considered excellent, 0.8-0.9 very good, 0.7-0.8 good, 0.6-0.7 sufficient, 0.5-0.6 bad, and less than 0.5 as not useful. The multicollinearity was examined using the variance inflation factor (VIF). A VIF value greater than 10 indicates the presence of a multicollinearity problem [17]. The AUC for this model was 0.722, indicating a good fit. The multicollinearity analysis showed that all variables had VIFs less than 5. This indicates that there was no evidence of multicollinearity among the independent variables.

## Discussion

Less than half of the Malaysian youths surveyed in this study were aware of the availability of SRH services. Similar low levels of awareness of SRH services have been reported in other Asian countries, such as Thailand and Sri Lanka, but slightly higher in a study from Ethiopia [18-20]. The low lifetime SRH services utilization rate found in this study could indicate that most study participants were unaware of SRH services within their community. This variation in awareness of SRH service availability may be attributed to cultural disparities and the inclusion of a broader representation of individuals from urban and rural regions without restricting participation to institutional settings, as was conducted in this study. The lack of awareness among participants in this study may reflect their lack of information and understanding of SRH.

Vaping is also linked to the utilization of SRH services. Vaping is increasingly common among youths in various parts of the world, and studies have suggested that vaping has a negative impact on sexual health, particularly on the genitourinary system [21]. Hence, this suggests higher utilization of SRH services among these vaping youths as they need to utilize SRH services for their various genitourinary issues.

In this study, the overall SRH services utilization in the past year was lower than those reported in studies conducted among youths aged 15-24 years in Myanmar [22] and Makassar, Indonesia [23]. However, utilization in the past year is slightly higher compared to another local study by Othman et al. in 2019, which reported only 6.9% utilization among four students in health facilities for SRH [5]. This difference could be because our study participants were from the older youth age group (18-24 years old). Older youth groups have more maturity and a wealth of sexual and reproductive health (SRH) experiences and knowledge, in contrast to younger adolescents who lack sexual experience and understanding of SRH services.

Marital status was one of the predisposing factors associated with the utilization of SRH services. Married couples are more likely to utilize SRH services, especially for pregnancy care. Studies have highlighted the possibility of misperceptions of the target audience of SRH services [24]. For example, younger youths may have the perception that SRH services, particularly family planning services, are only available to married couples.

Family as a source of information also emerged as a predisposing factor associated with SRH utilization. Previous studies have explored that individuals who discussed SRH issues with family were more likely to utilize SRH services compared to those who did not discuss such matters [25]. Confidence achieved during a discussion with family members on SRH issues may influence and promote higher utilization of SRH services [26]. This can be attributed to the self-assurance gained from discussing SRH challenges with family members, which, in turn, leads to the utilization of SRH services. However, as this study did not include confidence measurements, it is uncertain whether confidence plays a role in communication with family or friends.

This study also found that governmental agencies such as NPFDB in Malaysia as a source of information is associated with SRH utilization. Previous studies have suggested that visits to clinics managed by non-governmental organizations (NGOs) and NGO workers are associated with increased SRH utilization [27]. This calls for a more collaborative effort between government agencies (such as MOH and NPFDB) to work with NGOs to reach out to more youths regarding SRH. Support in the form of financial resources and training should be provided to NPFDB and NGOs, specifically regarding SRH, as they specialize in this area.

The comfort of utilizing SRH services emerged as one of the important factors in this study, including respondents reporting being too shy/embarrassed and lacking privacy during SRH service utilization. These findings align with another local study in which feeling embarrassed emerged as one of the main reasons for not accessing SRH services. Cultural sensitivities can still be rooted in the taboos surrounding the discussion of sex in Malaysian communities [5].

Addressing SRH as part of regular and overall health services could alleviate the impact of stigma toward risky behaviors associated with sexual health [5]. Youths should be informed that they are entitled to confidential services, and this information should be delivered as part of any SRH educational material. In addition, healthcare workers and personnel should undergo sensitization and stigma reduction training and be reminded of youths’ entitlement to confidential services.

In Malaysia’s current primary care setting, adolescent health services are available only to those aged 10-19 years [28]. There is a need to include the older age group of young people up to 24 years old. Youths above the age of 19 years are just as likely to prefer privacy and confidentiality. Furthermore, more collaboration and data integration between these youth-friendly services and STI clinics can be done. Currently, most patients are first screened at the outpatient clinic and then referred to the adolescent health clinic. Youths may be lost in this follow-up as they must return another day to be seen at the adolescent health clinic. The findings of this study suggest that youths prefer services that are easily accessible, less time-consuming, less stigmatized, and less costly. Therefore, the recommendation would be that once a patient fits the age criteria for adolescents and youth (10-24 years old), they may bypass the outpatient clinic and have direct access to youth-friendly clinics available within the primary care setting. This would reduce issues of loss of follow-up and be less time-consuming.

The ability of youths to self-purchase services in pharmacies is also an enabling factor for service utilization, as youths are more comfortable buying medicines themselves without approaching healthcare personnel. They may have received the information they needed online and through social media and are then able to purchase the medication themselves. Unfortunately, youths were not receiving optimal care as over-the-counter medicines were limited for SRH issues. The Malaysian MOH could consider increasing the ease of access to services and intervention at pharmacies, as done in other countries. For example, in Bolivia, where youth use pharmacies similarly, the government responded by training pharmacists to provide youth-friendly counseling. This provision of trained pharmacists has shown an increase in the utilization of SRH [29].

In this study, most respondents suggested a low perceived need for SRH services, and half were unaware of or uncomfortable utilizing SRH services. Participants who were diagnosed with SRH-related diseases were four times more likely to utilize SRH services compared to participants who had never been diagnosed with any SRH-related diseases.

Most Malaysians receive free or almost free healthcare services from the MOH, including SRH services throughout the preventive to treatment spectrum. The primary healthcare system has received widespread recognition as the best channel for disseminating SRH information and for preventing, detecting, and treating STIs, HIV, and various other SRH-related diseases [8]. SRH services for youths have been available in Malaysia since the 1990s by MOH and various other SRH providers in Malaysia, yet awareness of these services is generally still poor today. While Malaysia has made substantial progress in ensuring the provision of SRH services to the youths in the past decades, the services need to be expanded by integrating the needs of the current generation of youths into the public health system.

This study provides valuable baseline information for enhancing the present SRH services in various settings. Service providers need to change their advocacy mode for SRH information and provision to move away from outdated methods that do not work. Youths seek more help on online sites such as Google and social media. This calls for MOH to work with other stakeholders to provide an online platform, such as social media sites, where youths can seek help for SRH services. Studies have shown that youths spend more time on social media sites [30]. The more knowledge and awareness the youths have, and once they know they can seek help, the higher the likelihood of utilizing SRH services.

Strengths and limitations

This is the first nationwide study conducted in Malaysia among youths aged 18-24 years on SRH utilization adapting Andersen’s Healthcare Utilization Model. This model has helped identify the main factors related to SRH utilization from the youths’ perspective. This study provides baseline information and data on strategies and policies regarding SRH utilization for youths. These findings can be used by parents, educators, healthcare practitioners, and government agencies to develop prevention messages focusing on the importance of SRH utilization. Using the Internet and social media as a tool also increased the feasibility of this study because a low budget and a wider range of populations in the country could be reached.

A prominent limitation of this study is the fact that it was a cross-sectional study, limiting the examination of the cause-and-effect relationships between the variables of interest. Due to the sensitive nature of studies on reproductive issues, there is a possibility of social desirability bias, which can lead to an underestimation or overestimation of the extent to which reproductive health services are utilized. This web-based, cross-sectional design used convenience sampling and a social media platform. The findings may not be generalizable to the entire population of interest. This method may also lead to sampling bias and non-response bias. However, the potential bias was overcome by adding at least 20% more respondents to the sampling.

## Conclusions

Help-seeking behavior among the youths in Malaysia is poor; hence, most youths did not perceive the need to utilize SRH services in this study. The most probable cause of this outcome may be attributed to the challenges associated with the delicate nature of SRH, insufficient awareness of the availability of SRH services, and limited access to youth-friendly SRH services in different provider settings. This study emphasizes the significance and immediacy of empowering young people via focused education and involving SRH practitioners in delivering SRH services that are responsive to the needs of young people. Further research supported by qualitative methods should be conducted to better understand the cultural and service barriers that hinder the establishment of conducive environments for delivering accessible and effective reproductive healthcare to young individuals. This research should involve parents, teachers, and service providers.
